# Aging with a Liver Graft: Analysis of Very Long-Term Survivors after Liver Transplantation

**DOI:** 10.3390/jcm13041087

**Published:** 2024-02-14

**Authors:** Paolo De Simone, Jessica Bronzoni, Caterina Martinelli, Juri Ducci, Daniela Campani, Stefano Gitto, Piero Marchetti, Giandomenico Biancofiore

**Affiliations:** 1 Liver Transplant Program, University of Pisa Medical School Hospital, Via Paradisa 2, 56124 Pisa, Italy; 2 Department of Surgical, Medical, Biochemical Pathology and Intensive Care, University of Pisa, Via Savi 20, 56126 Pisa, Italy; 3 Hepatobiliary Surgery and Liver Transplantation, University of Pisa Medical School Hospital, Via Paradisa 2, 56124 Pisa, Italy; 4 Department of Pathology, University of Pisa Medical School Hospital, Via Paradisa 2, 56124 Pisa, Italy; 5 Internal Medicine and Liver Unit, University Hospital Careggi, Largo Brambilla 3, 50134 Florence, Italy; 6 Department of Experimental and Clinical Medicine, University of Florence, Piazza San Marco 4, 50121 Florence, Italy; 7 Diabetology Unit, University of Pisa Medical School Hospital, Via Paradisa 2, 56124 Pisa, Italy; 8 Department of Translational Research and New Technologies in Medicine and Surgery, University of Pisa, Via Savi 20, 56126 Pisa, Italy; 9 Intensive Care Unit, University of Pisa Medical School Hospital, Via Paradisa 2, 56124 Pisa, Italy

**Keywords:** liver transplantation, long term survival, aging, immunosuppression

## Abstract

Background: In Italy, data on long-term survivors after liver transplantation are lacking. Materials and Methods: We conducted a hybrid design study on a cohort of 359 adult recipients who received transplants between 1996 and 2002 to identify predictors of survival and the prevalence of co-morbidities among long-term survivors. Results: The actuarial (95% CI) patient survival was 96% (94.6–98.3%), 69% (64.2–73.6%), 55% (49.8–59.9%), 42.8% (37.6–47.8%), and 34% (29.2–38.9%) at 1, 5, 10, 15, and 20 years, respectively. The leading causes of death were hepatitis C virus recurrence (24.6%), extrahepatic malignancies (16.9%), infection (14.4%), and hepatocellular carcinoma recurrence (14.4%). The factors associated with the survival probability were younger donor and recipient ages (*p* = 0.001 and 0.004, respectively), female recipient sex (*p* < 0.001), absence of HCV (*p* < 0.01), absence of HCC (*p* = 0.001), and absence of diabetes mellitus at one year (*p* < 0.01). At the latest follow-up, the leading comorbidities were hypertension (53.6%), obesity (18.7%), diabetes mellitus (17.1%), hyperlipidemia (14.7%), chronic kidney dysfunction (14.7%), and extrahepatic malignancies (13.8%), with 73.9% of patients having more than one complication. Conclusions: Aging with a liver graft is associated with an increased risk of complications and requires ongoing care to reduce the long-term attrition rate resulting from chronic immunosuppression.

## 1. Introduction

Technical and scientific advancements have improved the outcomes of patients undergoing liver transplantation (LT), but long-term survival is still burdened by the complications of chronic immunosuppression [1]. The utilization of extended criteria donors (ECD), donors after cardiocirculatory death (DCD), and machine perfusion (MP) technology has increased in the evolving scenario of LT [2,3,4]. Additionally, strategies to reduce long-term attrition rates associated with immunosuppression, non-immunosuppressive co-medication, and recipient aging are widely advocated [5]. Although several international transplant centers have more than 20 years of clinical experience in LT, only a few studies have analyzed the long-term outcomes of LT recipients [6,7,8,9,10]. In Italy, there is a lack of data on the long-term outcomes of adult liver transplant recipients. Recent studies have provided estimates up to 10 years after transplantation [11]. To address this gap, we conducted a retrospective, comparative study on adult transplant recipients who survived for at least 20 years. Our aim was to identify clinical predictors of outcomes and showcase the long-term health conditions of the survivors.

## 2. Materials and Methods

### 2.1. Study Design

This was a retrospective, single-center study at an Italian National Health System (NHS)-based liver transplant center.

### 2.2. Population

The study involved all adult patients (≥18 years old) who underwent LT from deceased donors (DDLT) between January 1996 (when the transplant program began at our institution) and 31 December 2002. Patients were excluded from the analysis if they received a graft from a donor after cardiocirculatory death (DCD) or if they received combined transplantation.

### 2.3. Data Source

For the current study, we used data from the regional transplant authority (CRT, Centro Regionale Trapianti) and the prospectively maintained recipient database of our institution. The CRT database contains information on all donors, candidates on the waiting list, and transplant recipients, and ensures the accuracy, validity, and transparency of the data. The local ethics committee of the University of Pisa (Prot. 0036349/2020) approved all procedures.

### 2.4. Primary Outcomes

Our primary outcomes were post-transplant graft failure, patient death, and the incidence of hepatic and extrahepatic complications during the follow-up period. All measures were treated as time-to-event occurrences.

### 2.5. Secondary Outcome

Our secondary outcome was to evaluate the prevalence of long-term complications until December 2022.

### 2.6. Statistical Analyses

Descriptive statistics were calculated for the donor and recipient demographics and transplant characteristics. Patient characteristics were compared between groups using the appropriate statistical test, and survival data were censored at the time of graft failure, death, or the latest follow-up (December 2022). The significance level (*p*) was 5% (0.05). We utilized Cox proportional hazards models to assess the independent risks associated with post-transplant graft failure and patient mortality using the variables with a significance level ≤ 0.2 at univariable analysis and those with a reported impact on graft and patient outcome in the international literature: donor age and cause of death; recipient age; indication for transplantation; model for end-stage liver disease (MELD) score at transplantation; cold ischemia time (CIT); immunosuppression; diabetes mellitus at transplant and 1 year; and chronic kidney dysfunction (CKD). As complications within the first year after transplantation are more likely to be related to surgical/medical management, we performed sensitivity analyses that censored graft failure and death at 1 year after transplantation. The hazards of graft failure and death were again assessed using Cox proportional hazard models and adjusted as appropriate.

### 2.7. Special Considerations

During the study period (1996–2002), the transplant procedure, perioperative management, and immunosuppressive schedules changed according to technological advancements and scientific evidence. The University of Wisconsin (UW) perfusion solution was used until 2001, whereas Celsior^®^ (IGL, Lissieu, France) was utilized thereafter. Bypass, or the classical technique, was standard until 2017. De novo immunosuppression consisted of a triple regimen of microemulsion cyclosporine (CyA) (Neoral^®^, Novartis, Origgio (VA), Italy), steroids (S), and azathioprine (AZA) until 1999; CyA, S, and mycophenolate mofetil (MMF) until 2001; and a quadruple regimen with anti-CD25 (basiliximab, Simulect^®^, Novartis, Origgio (VA), Italy) since 2002. The use of tacrolimus (TAC) was initiated at our institution in 1999 and has become the standard de novo calcineurin inhibitor since 2009. The use of everolimus (EVR) (Certican^®^, Novartis, Origgio (VA), Italy) began in 2005 in maintenance schedules and in 2008 in de novo regimens. From 2002 onward, anti-CMV prophylaxis was administered to recipients without acquired immunity (D+/R− and D−/R− combinations).

### 2.8. Definitions and Cut-Offs

Cold ischemia time (CIT) was defined as the time from cross-clamping until the removal of the organ from the ice for implantation, and warm ischemia time (WIT) was defined as the time of ischemia during graft implantation. EAD was defined according to Olthoff et al. [12]. MELD scores at transplant were recalculated retrospectively based on available laboratory data. Rejection episodes were graded according to the BANFF scale [13]. HCV recurrence was diagnosed by liver biopsy in the presence of HCV-RNA. HBV infection recurrence was defined as the reappearance of HBsAg (± HBV DNA) in previously seroconverted patients, irrespective of liver function. Renal function was evaluated as the estimated glomerular filtration rate (eGFR) using the Modification of Diet in Renal Disease (MDRD)-4 formula. Renal dysfunction was defined as eGFR < 60 mL/min/1.73 m^2^ or a need for dialysis. Arterial hypertension was defined as the need for medication or a blood pressure of 140/90 mmHg at the following two visits. Diabetes mellitus was defined as the need for medication or a fasting plasma glucose level > 126 mg/dL at the following two visits. Dyslipidemia was defined as hypercholesterolemia of >220 mg/dL and/or hypertriglyceridemia of >200 mg/dL at the following two visits. Body mass index (BMI) was retrospectively derived from clinical charts for compensated patients during follow-up but not at the time of transplantation, as it was not possible to adjust values for the presence of ascites or edema. Obesity was defined as a BMI ≥ 30 kg/m^2^. For the current analysis, we excluded non-melanoma skin malignancies due to their negligible impact on patient survival.

## 3. Results

### 3.1. Demographics and Clinical Characteristics

During the study period, 375 procedures were performed on 367 patients (eight re-transplantations). Four patients were excluded from analysis due to their age at transplantation (<18 years) and four due to a combined liver and kidney transplantation. The final cohort included 359 patients. At a median (IQR) follow-up of 144.8 (204) months after transplantation, 123 (34.3%) recipients survived versus 236 (65.7%) who died.

The demographic and clinical characteristics of the study sample are shown in Table 1. The patients were predominantly male (66.6%) with a median (interquartile range (IQR)) age of 52.5 (13) years. The main indication for transplantation was HCV-related liver disease (54.3%), and hepatocellular carcinoma (HCC) was present in 42.1% of patients. The median (IQR) lab-MELD score at transplant (retrospective) was 13 (7). Diabetes mellitus (DM) was present in 12.2% of patients, CKD in 14.8%, and hypertension in 16.4% (Table 1). All recipients received a graft from a brain-dead donor (DBD) and a split liver was utilized in six cases (1.7%) only. The median (IQR) CIT was 536 (78) min. Eight (2.2%) patients were retransplanted due to primary non-function of the liver graft in six cases and hepatic artery thrombosis in two.

Immunosuppression at transplantation was based on CyA in 73.0% of patients, while anti-IL2R monoclonal antibody was used in 37.3% of cases (Table 1).

### 3.2. Patient and Graft Survival

**Figure 1:**
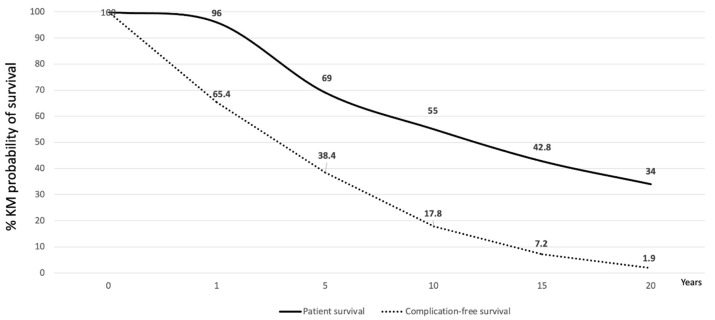
Kaplan–Meier probability of complication-free (dotted line) and patient survival (bold line) of the entire cohort of patients (#359) transplanted between 1996 and 2002.

Patient death and re-transplantation were considered graft loss. The actuarial (95% CI) patient survival of the overall study cohort was 96% (94.6–98.3%), 69% (64.2–73.6%), 55% (49.8–59.9%), 42.8% (37.6–47.8%), and 34% (29.2–38.9%) at 1, 5, 10, 15, and 20 years, respectively (Figure 1).

The actuarial (95% CI) graft survival was 93.8% (91.6–97.2%), 67.6% (62.2–71.5%), 54.3% (49.6–59.3%), 42.1% (37.3–47.4%), and 33.8% (29.0–38.4%) at 1, 5, 10, 15, and 20 years, respectively.

### 3.3. Causes of Death

After a median (IQR) follow-up of 144.8 (204) months, 236 (65.7%) patients had died. The causes of death are presented in Table 2. Recurrent HCV-related liver dysfunction was the most common cause of death, accounting for 24.6% of all the deaths. This was followed by extrahepatic malignancies (16.9%), infection (14.4%), HCC recurrence (14.4%), and major cardiovascular events (MACE) (10.6%). Half of all deaths occurred within 5.5 years after the transplant.

### 3.4. Rejection

The incidence of treated and biopsy-proven acute rejection (t/BPAR) is shown in Table 2. Seventy-seven (21.4%) patients presented with at least one episode of t/BPAR; the proportion of patients with more than one episode was 3.9%. Half of all rejection episodes occurred within 6 months after transplantation (median (IQR) 11 (3) weeks).

### 3.5. Health Conditions of Long-Term Survivors

As of December 2022, a cross-sectional analysis of the 123 survivors revealed that the most frequent co-morbidities were hypertension (53.6%), obesity (18.7%), DM (17.1%), hyperlipidemia (14.7%), CKD (14.7%), extrahepatic malignancies (13.8%), and MACE (13.0%) (Table 3). Most patients (73.9%) experienced more than one complication.

Maintenance immunosuppression consisted of TAC monotherapy in 38.2% of the patients, CyA monotherapy in 29.2%, EVR monotherapy in 13.8%, TAC + EVR in 8.9%, and TAC + MMF (or AZA) + S in 2.4% (Supplementary Table S1).

### 3.6. Complication-Free Survival

Figure 1 shows the Kaplan–Meier probability (95% CI) of survival without any complication/comorbidity during the follow-up period. At 1, 5, 10, 15, and 20 years this was 65.4% (60.2–70.3%), 38.4% (33.4–43.7%), 17.8% (14.1–22.2%), 7.2% (4.8–10.5%), and 1.94% (0.85–4.15%).

### 3.7. Predictors of Survival

The univariable analysis showed that the chances of surviving after transplant were higher for recipients of younger grafts (*p* = 0.0009), younger patients (*p* < 0.0001), female patients (*p* = 0.005), those with non-HCV liver disease as the reason for transplantation (*p* < 0.0001), no presence of HCC at transplant (*p* = 0.001), a lower laboratory MELD score (*p* = 0.002), the use of anti-IL2R as an induction agent (*p* < 0.0001), TAC as the primary immunosuppressant (*p* < 0.0001), and no occurrence of DM one year after transplant (*p* = 0.01) (Table 4**)**. The Cox proportional hazards analysis revealed that several independent factors were associated with the survival probability (Table 5). These factors associated with successful liver transplantation include younger donor and recipient ages (*p* = 0.001 and 0.004, respectively), female recipient sex (*p* < 0.001), the absence of HCV (*p* < 0.01), the absence of HCC (*p* = 0.001), and the absence of DM at one year (*p* < 0.01).

## 4. Discussion

To our knowledge, this is the first report of adults surviving at least 20 years after liver transplant in Italy. Despite the expanding practice and improved outcomes of pediatric and adult LT [14,15,16,17,18], there is a lack of information on long-term survivors in our country. The only long-term reports are the ones from Lai et al. and Cussa et al. on adult and pediatric recipients, respectively [11,19]. We conducted this single-center study to address this gap and provide data for comparison with international benchmarks. It is crucial to research the factors associated with long-term survival to ensure the optimal use of donated organs, especially considering the increasing age of utilized organs and recipients. The trend can be attributed to various factors, such as the shortage of organs, epidemiological developments, and the emergence of novel technologies like ex-situ machine perfusion (MP) [4].

Comparing long-term results across national and international series is challenging due to biases related to recipients, donors, indications for transplantation, eras, and the scarcity of publications [20]. Most deaths occur within 5 years after transplantation, and while early mortality is mainly related to the severity of the native liver disease, the recipient’s health conditions, the quality of the liver grafts, and surgery-related complications, death beyond 1 year is due to an increased risk of cancer, cardiovascular events, infection, and graft failure [20]. Most studies concentrate on a limited period and number of factors for patient survival after transplantation, which may lead to incomplete modeling due to insufficient information. Additionally, assumptions of linearity and independence among predictor variables in existing post-transplant outcome prediction studies may not be valid, especially in the long term [20]. One recent approach is the application of deep learning algorithms on longitudinal data of large data sets. In a study on 42,146 LT recipients from the Scientific Registry of Transplant Recipients (SRTR) in the USA and the University Health Network (UHN) database in Ontario, Canada, the variables associated with long-term graft-related mortality were HCV as an indication to transplantation, donor age, rejection after transplantation, and post-transplantation diabetes, hypertension, and renal insufficiency [20]. This machine learning approach to modeling long-term survival after LT is currently unavailable for our national data, and the approach we can use to set our experience against an international benchmark is through a comparative analysis of single-center studies.

In the only Italian study on long-term LT survivors, 491 patients were included, with a median survival of 10.4 years. Out of the study cohort, there were 263 long-surviving (LS) patients, which accounts for 53.6% of the total. Among the 491 patients, 134 (27.3%) had a follow-up time of 15 years or more, while 56 (11.4%) had a follow-up of 20 years or more. The patient with the longest follow-up had a duration of 33.3 years [11]. The predictors of a shorter survival in the authors’ experience were both recipient (OR = 1.02, 95.0% CI = 1.01–1.04; *p* = 0.01) and donor ages (OR = 1.01, 95.0% CI = 1.00–1.03; *p* = 0.03), being transplanted during the eighties and nineties (1982–1991 OR = 6.46, 95.0% CI = 3.05–13.68; *p* < 0.0001; 1992–2001 OR = 2.63, 95.0% CI = 1.67–4.15; *p* < 0.0001) versus 2002–2012. Being transplanted with a UNOS status 1 or 2A also correlated with poor survival (OR = 2.62, 95.0% CI = 1.65–4.16; *p* < 0.0001) [11].

Our experience is not entirely comparable because it began in 1996, later than Lai’s start date of 1982, and comes from a center that has performed more transplants. While some risk factors regarding the donor and recipient’s age were confirmed, other predicting variables were not. This can be accounted for by the different donor and recipient populations included in the centers’ experiences, the relatively different proportions of urgent recipients, surgical techniques (concerning caval sparing), transplant eras (our experience started in 1996), and the different immunosuppressive regimens implemented at our center (regarding use of induction agents, i.e., anti-IL2R). However, our 10-year patient survival rate (55%, 95% CI = 49.8–59.9%) matches favorably with the one reported by Lai et al. (53.4%), underpinning the transferability of our experience to the national scale.

Several studies show significant variability in long-term data from European and American centers. In one of the largest single-center experiences on 4000 recipients who were followed-up on for almost two decades after transplantation, Jain et al. showed 1, 5-, 10-, 15-, and 18-year patient survival rates of 79%, 67%, 57%, 50%, and 48%, respectively [6]. Although their experience included pediatric recipients (12%), survival was significantly better in children, female recipients, and patients who received transplants after 1990 [6]. The recurrence of disease, malignancies, and age-related complications were the major long-term graft loss factors [6]. A smaller series from the USA on 293 patients between 1984 and 1988, including 114 children, showed a 20-year actuarial patient survival rate of 52% (42% for the graft) [7]. The factors associated with long-term survival included recipient age <18 (*p* = 0.01), nonurgent LT (*p* = 0.01), no retransplantation (*p* = 0.02), female gender (*p* = 0.03), the absence of biliary complications (*p* = 0.04), and short total ischemia time (*p* = 0.05) [7].

In a German single-center experience on 313 patients transplanted between 1988 and 1992, patient and graft survival at 20 years were higher than in our series (i.e., 52.5% and 46.6%, respectively) [8]. The primary indication (*p* < 0.001), age (*p* < 0.001), gender (*p* = 0.017), impaired renal function at 6 months (*p* < 0.001), and retransplantation (*p* = 0.034) had a significant impact on patient survival [8]. The leading causes of death were recurrent disease (21.3%), infection (20.6%), and de novo malignancy (19.9%) [8].

In a Spanish experience by Dopazo et al. on 132 patients who received 151 deceased-donor transplants between 1988 and 1993, 31% (*n* = 41) of recipients died within the first year, with a further 31% (*n* = 41) at 5 years, and a further 16.7% (*n* = 22) were dead at 20 years, leaving only 21% (*n* = 28), approximately one-in-five of the recipients, surviving to 20 years. Infections were the leading cause of death within 1 year (32%) and between 1 and 5 years (25%), while HCV recurrence was the main cause of death beyond 5 years (22%) [10]. The factors with an impact on long-term patient survival were HCC (*p *= 0.049), pre-transplant renal dysfunction (*p *= 0.043), a long warm ischemia time (*p *= 0.016), post-transplant DM (*p *= 0.001), and liver dysfunction (*p *= 0.05) at 1 year [10]. A recent retrospective analysis of 3682 long-term (>5 years) survivors at three high-volume centers from the USA, Spain, and the UK showed that the predictors of long-term mortality were older recipients and older donors, malignancy, cardiovascular disease, and dialysis underpinning the need for a strict follow-up and appropriate medical interventions [21].

Despite differences related to eras and the historical impact of HCV, taken together, all long-term reports highlight the burden of comorbidities related to patients’ aging and immunosuppression, including cardiovascular, metabolic, renal, and oncological complications. Our study observed that almost all patients who survived 20 years post-transplantation had at least one comorbidity, and 73.9% had multiple comorbidities. Similar values were reported by Rubin et al. [9], with most deaths related to recurrent HCV graft disease, followed by de novo tumors and cardiovascular events. In their experience, the 1-, 3-, 5-, and 10-year cumulative rates of cardiovascular events and de novo tumors above the baseline were 2%, 5%,10% and 17% and 1%, 3%, 6%, and 13%, respectively [9]. These values are consistent with the incidence of complications observed in our cohort and the prevalence of complications/comorbidities observed at the latest follow-up in the group of long-term survivors.

The international literature has well documented our cohort’s long-term survival predictors. Although the significance of HCV is now historical, several variables remain relevant in everyday clinical practice. These variables include donor and recipient ages, HCC in the native liver, and metabolic complications before or after transplantation. The early outcomes of transplant surgeries are more dependent on the graft quality, the surgery itself (measured by CIT and WIT), and the clinical severity of the recipient. To note, all these risk factors translate into an increased incidence of infection-related mortality early on after surgery. In the long term, the outcomes of LT are influenced by the demographic characteristics of the patients (such as age and sex), the indication for transplantation (such as HCV and HCC), and any complications that may occur in the first few years after the surgery, especially diabetes mellitus, chronic kidney dysfunction, and de novo malignancies. Most of these latter comorbidities are induced by chronic immunosuppression, but the role of aging and age-related immune dysfunction should not be underestimated. The study by Dopazo et al. [10] highlighted the predictive role of DM one year after transplantation. This was confirmed in our experience, and we advocate that DM may be an end-point surrogate for pre- and clinical studies on novel immunosuppressive agents and therapeutic technologies in the near future. In line with our findings, some published studies have shown that female recipients have a higher probability of long-term survival [6,7,8]. This should be analyzed in the context of the higher life expectancy rates of females, as well as the differences in the prevalence of liver diseases like hepatitis C between male and female patients.

It is necessary to consider the specific constraints of our research and validate the findings on a larger scale and a more recent time frame. All the predictive factors for long-term survival (i.e., the age of the donor, male sex, the age of the recipient, HCV infection, the presence of HCC, and the diagnosis of diabetes 1 year after the transplant) cannot be used for modifying decisions in clinical practice. However, the practice of using older donor grafts is expanding [2,3], including older recipients, while HCV infection has lost its prognostic role [18]. Possible solutions to these issues might be the implementation of donor-to-recipient allocation algorithms accounting for donor and recipients’ ages and mitigating transplant-related ischemia/reperfusion injury and its negative impact on graft function [3]. Post-transplant recipients should undergo close metabolic monitoring and reduce risk factors for post-transplant diabetes through measures such as adjusting immunosuppressive and non-immunosuppressive drugs, following a healthy diet, and engaging in regular exercise [21].

To better understand the impact of comorbidities on long-term survivors compared to national averages, a recent study found that 6.6% of the Italian population had diabetes in 2022, ranging from 1% for people aged 25–34 years to 21.6% for those aged 75 years or older [22]. Compared to the 31% hypertension prevalence in individuals aged 35–74 years in Italy [23], the latest follow-up in our study reported a prevalence of 53.6%. According to an Italian epidemiological study, the prevalence rates of CKD for males and females aged 35–79 years were 7.5% and 6.5%, respectively [24], whereas the prevalence rate was 14.7% in our series. Finally, the prevalence rate of malignancies in our country in 2020 was 2.0% (i.e., 1,230,693 new prevalent cases in the preceding 5 years) [25] against a 13.8% prevalence of extra-hepatic malignancies at the latest follow-up in our series.

Our study has several limitations. First, its retrospective design does not always allow for the granular information that real-practice clinical studies need to produce clinically transferable data. Additionally, it focused on the initial experience at our center and might inevitably be biased by a learning curve effect in terms of technical refinements, patient selection, and post-transplant therapeutic strategies. Finally, the quality of chronic care provided at our center over a patient’s lifetime horizon changed during the follow-up period and might have affected the individual patient’s probability of post-transplant survival.

In conclusion, based on our results, the probability of long-term survival after liver transplantation is higher for younger recipients who receive younger grafts, female patients, those with no HCC in the explant liver, and those without DM in the post-transplant course. Additionally, aging with a liver graft is associated with an increased risk of metabolic, cardiovascular, and oncologic comorbidities and requires a chronic care model to reduce the long-term attrition rate resulting from chronic immunosuppression.

## Figures and Tables

**Table 1:** Demographic and clinical characteristics of interest in the study population.

Variable	Patients (#359)
Male sex, *n* (%)	239 (66.6)
Age at transplant (median, IQR), years	52.5 (13)
Indication for transplant, *n* (%)	
HCV	195 (54.3)
HBV (±HDV)	71 (19.8)
HCV-HBV (±HDV)	17 (4.3)
Alcohol	29 (8.1)
MASLD	13 (3.6)
Autoimmune	16 (4.4)
ALF	9 (2.5)
Other	9 (2.5)
Co-existing HCC	151 (42.1)
Lab-MELD at transplant (median, IQR) *	13 (7)
DM at transplant, *n* (%) *	44 (12.2)
CKD at transplant, *n* (%)	53 (14.8)
Hypertension at transplant, *n* (%)	59 (16.4)
DBD, *n* (%)	359 (100)
Donor age, median (IQR)	68.5 (15)
Trauma, *n* (%)	103 (28.7)
Split graft, *n* (%)	6 (1.7)
CIT, median (IQR) (min)	536 (78)
Re-transplantation, *n* (%)	8 (2.2)
PNF/EAD	6 (1.7)
HAT	2 (0.6)
Immunosuppression at transplant, *n* (%)	
CyA-based	262 (73.0)
TAC-based	97 (27.0)
With anti-IL2R	134 (37.3)

Note: ALF, acute liver failure; CIT, cold ischemia time; CKD, chronic kidney failure; CyA, cyclosporine; DBD, donors after brain death; DM, diabetes mellitus; EAD, early allograft dysfunction; HAT, hepatic artery thrombosis; HBV, hepatitis B virus; HCV, hepatitis C virus; HCC, hepatocellular carcinoma; HDV, hepatitis delta virus; IQR, interquartile range; IL2R, interleukin-2-receptor; MASLD, metabolic dysfunction-associated steatotic liver disease; MELD, model for end-stage liver disease; PNF, primary nonfunction; TAC, tacrolimus; * retrospectively available for 321 (89%) patients.

**Table 2:** Results.

Variable	Patients (#359)
Follow-up (median, IQR), months	144.8 (204)
DM at 1 year after transplant, *n* (%)	76 (21.1)
CKD at 1 year after transplant, *n* (%)	32 (8.9)
Hypertension at 1 year after transplant, n (%)	53 (14.7)
Death, *n* (%)	236 (65.7)
HCV recurrence, *n* (%)	58 (24.6)
Extra-hepatic malignancies, *n* (%)	40 (16.9)
PTLD	24 (10.1)
Lung	8 (3.4)
Colon	5 (2.1)
Breast	2 (0.8)
Occult primary	1 (0.4)
Infection, *n* (%)	34 (14.4)
HCC recurrence, *n* (%)	34 (14.4)
MACE, *n* (%)	25 (10.6)
Initial poor function of the graft, *n* (%)	13 (5.5)
Native liver disease recurrence other than HCV, *n* (%)	10 (4.2)
Ischemic cholangiopathy, *n* (%)	8 (3.4)
Intraoperative, *n* (%)	8 (3.4)
Cholangiocarcinoma recurrence, *n* (%)	6 (2.5)
t/BPAR, *n* (%)	77 (21.4)
One episode	63 (17.5)
Two episodes	13 (3.6)
>two episodes	1 (0.3)

Note: CKD, chronic kidney dysfunction; DM, diabetes mellitus; HCC, hepatocellular carcinoma; HCV, hepatitis C virus; MACE, major cardiovascular event; PTLD, post-transplant lymphoproliferative disease; t/BPAR, treated and biopsy-proven acute rejection.

**Table 3:** Prevalence of co-morbidities in survivors at data censor (December 2022).

Variable	*n* * (%) (#123)
Hypertension	66 (53.6)
DM	21 (17.1)
Hyperlipidemia	12 (14.7)
CKD	12 (14.7)
Extra-hepatic malignancies	17 (13.8)
PTLD	9 (7.3)
Melanoma	3 (2.4)
Laryngeal	3 (2.4)
Breast	1 (0.8)
Anal	1 (0.8)
MACE	16 (13.0)
Neurologic	5 (4.1)
VBDS/chronic rejection	3 (2.4)
Patients with >1 complication, *n* (%)	91 (73.9%)

Note: CKD, chronic kidney disease; DM, diabetes mellitus; HCC, hepatocellular carcinoma; MACE, major cardiovascular event; VBDS, vanishing bile duct syndrome. * As per patient with condition.

**Table 4:** Univariable analysis of predictors of survival.

Variable	Survivors (#123)	Died (#236)	*p*
Follow-up (median, IQR), months	255.3 (12)	64.6 (115.5)	<0.0001
Male sex, *n* (%)	70 (56.9)	169 (71.6)	0.005
Age at transplant (median, IQR), years	43 (14)	55 (14)	<0.0001
Indication for transplant, *n* (%)			
HCV	50 (40.6)	145 (61.4)	<0.000
HBV (±HDV)	30 (24.4)	41 (17.3)	0.09
HCV-HBV (±HDV)	6 (4.9)	11 (4.7)	0.92
Alcohol	8 (6.5)	21 (8.9)	0.42
NAFLD	5 (4.1)	8 (3.4)	0.74
Autoimmune	12 (9.7)	4 (1.7)	<0.001
ALF	6 (4.9)	3 (1.3)	0.08
Other	6 (4.9)	3 (1.3)	0.08
HCC	38 (30.9)	113 (47.8)	0.001
Lab-MELD at transplant (median, IQR) *	12 (7)	15 (8)	0.002
DM at transplant, *n* (%) *	12 (9.7)	32 (16.5)	0.29
CKD at transplant, *n* (%)	16 (13.0)	37 (15.7)	0.49
Hypertension at transplant, *n* (%)	21 (17.1)	38 (16.1)	0.81
DM at 1 year after transplant, *n* (%)	18 (14.6)	58 (25.8) **	0.01
CKD at 1 year after transplant, *n* (%)	11 (8.9)	21 (9.3) **	0.9
Hypertension at 1 year after transplant, *n* (%)	18 (17.1)	35 (15.5)	0.81
TAC as primary immunosuppressant, *n* (%)	50 (40.7)	47 (19.9)	<0.0001
Use of anti-IL2R, *n* (%)	91 (73.9)	43 (18.2)	<0.0001
DBD, *n* (%)	190 (100)	246 (100)	0.99
Donor age, median (IQR) (years)	68 (16)	73 (15)	0.0009
Trauma, *n* (%)	39 (31.7)	64 (27.1)	0.36
Split graft, *n* (%)	1 (0.5%)	5 (2.0%)	0.35
CIT, median (IQR) (min)	523 (76)	542 (84)	0.08

Note: CIT, cold ischemia time; CKD, chronic kidney failure; CyA, cyclosporine; DBD, donors after brain death; DM, diabetes mellitus; EAD, early allograft dysfunction; HAT, hepatic artery thrombosis; HBV, hepatitis B virus; HCV, hepatitis C virus; HCC, hepatocellular carcinoma; HDV, hepatitis delta virus; IQR, interquartile range; IL2R, interleukin-2-receptor; MASLD, metabolic dysfunction-associated steatotic liver disease; MELD, model for end-stage liver disease; PNF, primary nonfunction; TAC, tacrolimus; * retrospectively available for 321 (89%) patients; ** out of 225 patients surviving ≥1 year.

**Table 5:** Multivariable analysis of predictors of long-term survival.

Variable	HR	*p*
Donor age	1.09	0.032
Trauma as cause of donor’s death (yes/no)	1.06	0.78
CIT	1.04	0.67
Recipient male sex (yes/no)	1.15	<0.001
Recipient age at transplant	1.09	0.004
Recipient HCV (yes/no)	1.22	<0.01
Recipient autoimmune disease	1.03	0.56
HCC (yes/no)	1.17	0.001
Lab-MELD at transplant	1.04	0.74
DM at transplant (yes/no)	1.07	0.063
DM at 1 year after transplant (yes/no)	1.12	<0.01
CKD at transplant (yes/no)	1.06	0.064
TAC as primary immunosuppressant (yes/no)	0.92	0.071
Use of anti-IL2R (yes/no)	0.91	0.062

Note: the reference categories for continuously distributed variables were: median donor age (68.5 years); median CIT (536 min) and median lab-MELD (13). CIT, cold ischemia time; CKD, chronic kidney dysfunction; DM, diabetes mellitus; HCC, hepatocellular carcinoma; HCV, hepatitis C virus; IL-2R, interleukin-2 receptor; MELD, model for end-stage liver disease; TAC, tacrolimus.

## Data Availability

Data supporting the current research are available upon reasonable request.

## Supplementary Materials

The following supporting information can be downloaded at: https://www.mdpi.com/article/10.3390/jcm13041087/s1, Supplementary Table S1: Maintenance immunosuppression in survivors.
