# Supplementary material for: Aging with a Liver Graft: Analysis of Very Long-Term Survivors after Liver Transplantation
Source: J Clin Med. 2024 Feb 14;13(4):1087. doi: 10.3390/jcm13041087 (PMC10889074; doi:10.3390/jcm13041087)
Supplement: Supplementary file 1 [file jcm-13-01087-s001.zip › jcm-2842953 supplementary.pdf]

Supplementary Table S1: Maintenance immunosuppression in survivors.

**Regimen**	**n* (%)**<br>**(#123)**
TAC monotherapy	47 (38.2)
ME-CyA monotherapy	36 (29.2)
EVR monotherapy	17 (13.8)
TAC + EVR	11 (8.9)
TAC + MMF (AZA)	9 (7.3)
TAC + MMF (AZA) + S	3 (2.4)

Note: AZA, azathioprine; CyA, cyclosporine; EVR, everolimus; ME, microemulsion; MMF, mycophenolate mofetil; S, steroids; TAC, tacrolimus.
